# Stereotactic radiosurgery for glioblastoma: retrospective analysis

**DOI:** 10.1186/1748-717X-4-11

**Published:** 2009-03-17

**Authors:** Tithi Biswas, Paul Okunieff, Michael C Schell, Therese Smudzin, Webster H Pilcher, Robert S Bakos, G Edward Vates, Kevin A Walter, Andrew Wensel, David N Korones, Michael T Milano

**Affiliations:** 1 Department of Radiation Oncology, University of Rochester Medical Center, Rochester, NY 14642, USA; 2 Department of Neurosurgery, University of Rochester Medical Center, Rochester, NY 14642, USA; 3 Department of Medicine, Division of Oncology, University of Rochester Medical Center, Rochester, NY 14642, USA; 4 Department of Pediatrics, University of Rochester Medical Center, Rochester, NY 14642, USA; 5 Department of Neurology, University of Rochester Medical Center, Rochester, NY 14642, USA

## Abstract

**Purpose:**

This retrospective study was done to better understand the conditions for which stereotactic radiosurgery (SRS) for glioblastoma may be efficacious.

**Methods:**

Between 2000 and 2007, 33 patients with a pathological diagnosis of glioblastoma received SRS with the Novalis^® ^Shaped Beam Radiosurgery system. Eighteen patients (54%) underwent salvage SRS for recurrence while 15 (45%) patients received upfront SRS following standard fractionated RT for newly diagnosed glioblastoma.

**Results:**

There were no RTOG grade >2 acute side effects. The median survival after SRS was 6.7 months (range 1.4 – 74.7). There was no significant difference in overall survival (from the time of initial diagnosis) with respect to the timing of SRS (p = 0.2). There was significantly better progression free survival in patients treated with SRS as consolidation versus at the time of recurrence (p = 0.04). The majority of patients failed within or at the margin of the SRS treatment volume (21/26 evaluable for recurrence).

**Conclusion:**

SRS is well tolerated in the treatment of glioblastoma. As there was no difference in survival whether SRS is delivered upfront or at recurrence, the treatment for each patient should be individualized. Future studies are needed to identify patients most likely to respond to SRS.

## Introduction

Glioblastoma is an aggressive primary brain tumor with poor outcome. Fractionated radiation therapy is the primary adjuvant treatment following resection or biopsy [1]. The addition of concurrent (with radiation) and adjuvant temozolomide has recently been shown to significantly improve survival in glioblastoma, and is now considered to be standard of care following resection [2-4]. However, the inability to adequately locally control disease, either upfront or at recurrence, continues to be a major challenge for this highly infiltrative tumor.

There appears to be a dose response to radiotherapy for glioblastoma up to about 60 Gy. In late 1970s, retrospective analyses of previous randomized trials by the Brain Tumor Study Group established a dose-response between 50 – 60 Gy for glioblastoma [5]. Another randomized study by Medical Research Council confirmed that there is an improvement in median survival from 9 months to 12 months when adjuvant radiation dose was increased from 45 Gy to 60 Gy [6]. Several prospective dose escalation trials, some utilizing hyperfractionation or accelerated fractionation regimens have suggested a possible dose response relationship for glioblastoma, though no randomized trials have shown any survival benefit over standard fractionated 60 Gy [1,7-15]. The Radiation Therapy Oncology Group (RTOG) conducted a phase III randomized study (RTOG 93-05) for newly diagnosed glioblastoma to assess whether the addition of upfront stereotactic radiosurgery (SRS) improves patient outcome; the results suggested that an SRS boost does not significantly improve disease control or patient survival [16].

Regardless of the initial treatment, glioblastoma recurrences predominantly occur at the site of the initial tumor [17-20]. For recurrent glioblastoma, the prognosis is usually grim. Repeat surgery when feasible and chemotherapy are often used for salvage of recurrent glioblastoma. Re-irradiation with standard fractionated radiotherapy is feasible, particularly with modern radiation techniques, but often considered risky because of the cumulative dose to a large volume of normal brain structures [21]. SRS, by virtue of its improved set-up accuracy, allows for a reduction in the volume receiving the prescribed dose, and can therefore be advantageous over standard radiotherapy. The role of SRS in this setting has not been tested in a prospective randomized study. Several retrospective series have demonstrated a median survival on the order of 7–11 months, albeit with a of selection bias favoring patients who are amenable to SRS [22-30]. We undertook this retrospective analysis to look at the SRS dosimetry treated with Novalis Shaped Beam Radiosurgery, as well as the outcome of all treated patients.

## Clinical Materials and Methods

The University of Rochester has been treating intracranial lesions with SRS since December 1992 and has been using the Novalis Shaped Beam Surgery system with BrainLAB planning software since 2000.

The charts of 33 patients with a pathological diagnosis of glioblastoma (WHO grade IV) who underwent SRS treatment between November 2000 and April 2007 were retrospectively reviewed. The study was approved by the University of Rochester Research Subjects Review Board.

### Patient Selection

Patients were selected to be amenable to SRS if their enhancing lesions were <4 cm in size. The decision for a patient to undergo SRS following fractionated radiotherapy for newly diagnosed glioblastoma was at the discretion of the treating physicians, including the radiation oncologist, medical oncologist and the neurosurgeon. The majority of patients who received upfront SRS were either treated prior to the published result of RTOG-93-05 or as part of an ongoing protocol in which SRS was allowed [31]. The decision for patients to undergo salvage SRS was also at the discretion of the treating physicians.

### Radiosurgery Treatment

SRS was delivered with the Novalis linear accelerator (BrainLAB A.G., Heimstetten, Germany), equipped with micromultileaf collimators (MLC), using 6 MV photons. The tungsten MLC leaves at the center of the field are 3 mm in thickness. A BrainLAB stereotactic head frame (BrainLAB A.G., Heimstetten, Germany) was used for immobilization during the computerized tomography (CT) scan and during treatment. A Novalis localizer frame (BrainLAB A.G., Heimstetten, Germany) was attached during the CT scan. CT and magnetic resonance imaging (MRI) scans were used to delineate the target and normal structures. Lesion enhancement seen on the Gadolinium-T1 weighted MRI images were defined as GTV. The GTV was expanded by 0–1 mm to generate the PTV. BrainLAB planning software (BrainLAB A.G., Heimstetten, Germany) was used to generate a treatment plan. The prescribed isocenter dose was generally 10–20 Gy, extrapolating from the RTOG protocol 90-05 guidelines in which prescribed dose was dependent upon target volume [32]. The maximum prescribed dose was based on size of the lesion and the proximity of critical structures. We generally did not prescribe the isocenter dose >20 Gy. Generally, the ≥80% isodose line covered ≥99% of the PTV. The dose constraints for critical structures were: brain stem <10 Gy; optic chiasm and optic nerves <8 Gy; normal brain (brain minus GTV) <14 Gy. All patients received 10 mg of intravenous decadron prior to their SRS and were kept overnight in the hospital for observation.

### Follow-up

Following SRS, all patients were followed at 6 weeks and thereafter every 2–3 months. For each follow-up examination, every patient underwent a contrast enhanced MRI scan and a neurological evaluation. MR-spectroscopy, MRI perfusion and/or PET imaging were performed as needed to help to distinguish radiation necrosis from tumor progression.

### Statistical analysis

Both overall survival and progression free survival were our endpoint analysis. Using the Kaplan-Meier method, overall survival was calculated from two different time points: from the initial diagnosis and from the time of SRS. Progression free survival was calculated from the time of SRS until tumor recurrence, tumor progression or death, which ever occurred first. All statistical analysis was performed using statistical software Stata 9.1.

## Results

### Patient Characteristics

The patient and tumor characteristics are shown in Table 1. All patients had a Karnofsky performance status of ≥70. The median age at the time of diagnosis was 57.8 years (range 33–81 years). All patients received external beam radiation, delivered to a median dose of 60 Gy (range 50–64 Gy) with 1.8 – 2 Gy per fraction. Six patients were treated with accelerated radiation (64 Gy, 1.6 Gy twice daily) as part of a University of Rochester pilot protocol[31] Sixteen patients (48%), received temozolomide concurrently during their fractionated radiotherapy, 2 patients received other concurrent chemotherapy and 15 (45%) patients received no concurrent chemotherapy. The majority of patients (85%) received adjuvant chemotherapy after radiation while 15% received no adjuvant chemotherapy. Adjuvant chemotherapy included single agent temozolomide or BCNU in most patients. In a few patients, other drugs, such as irinotecan or etoposide were given in combination with BCNU or temozolomide.

**Table 1:** Patient Characteristics

**Variables**	**Number**	**%**
Gender		
Male	19	(57)
Female	14	(43)
Age ≤ 50	9	(27)
Age > 50	24	(73)
Extent of surgical resection		
Gross total resection	8	(24)
Subtotal resection	9	(27)
Biopsy	16	(48)
Tumor location		
Frontal	12	(36)
Temporal	9	(19)
Parietal	6	(27)
Multifocal	6	(18)

A total of 18 patients underwent SRS at recurrence, 14 underwent SRS upfront as a consolidation and 1 patient underwent SRS upfront as consolidation as well as at the time of recurrence (12 months after the first SRS). The latter patient, for the purpose of data analysis, was included in the group of patients undergoing upfront consolidative SRS. Another patient received two salvage SRS procedures, to the same location, for two chronologically separate recurrences (8 months apart). In the 18 patients treated for recurrence, the median interval from diagnosis to SRS was 12.1 months (4.1 – 44.4); while in the other 15 patients it was 1.3 months (0.4 – 7.6). For patients who received SRS at recurrence, the median interval from the fractionated to the SRS was 9.1 months (range 1.8 – 40.2 months).

Table 2 summarizes the SRS dosimetric analysis. The median dose at the isocenter was 14 Gy. The median and maximum volume for consolidative and salvage SRS were not significantly different (p = 0.2)

**Table 2:** Radiation treatment parameters

	Median	Minimum	Maximum
Dose @ isocenter (Gy)*			
All patients	14	6*	20
Upfront SRS	13	6*	20
SRS at recurrence	15	9	20
Peripheral dose (%)			
All patients	80	40	100
Upfront SRS	80	50	100
SRS at recurrence	80	40	90
Number of isocenters			
All patients	2	1	7
Upfront SRS	1	1	3
SRS at recurrence	2	1	7
Number of beams/arcs			
All patients	12	6	32
Upfront SRS	12	7	24
SRS at recurrence	12	6	32
GTV (ml)			
All patients	9.2	0.2	85.4
Upfront SRS	13.2	1.5	85.4
SRS at recurrence	8.4	0.2	32.2
Upfront fractionated RT dose (all patients)	60	50	65

* because of worsening neurological symptoms, large treatment volume and critical location, one patient was prescribed an unusually low isocenter dose of 6 Gy.

One patient who received 6.4 Gy at the isocenter underwent SRS following fractionated radiotherapy for tumor progression and worsening neurological symptoms. The lesion was located in the left parietal lobe close to the motor strip and had a cystic component as well as an enhancing component of the lesion. The entire area was treated using 2 isocenters. Given the patient's worsening neurological symptoms, and close proximity of the motor strip, he was treated with an unusually low dose.

### Toxicity

The RTOG toxicity criteria were used. There was no grade >2 acute toxicity. One patient developed grade 4 late-toxicity. This patient was initially treated with accelerated radiotherapy (64 Gy in twice-daily 1.6 Gy fractions) with concurrent temozolomide, followed by an SRS boost and adjuvant temozolomide. His post-SRS MRI scans at 2 and 4 months showed enlarging tumor with altered enhancement and decreased blood perfusion; magnetic resonance spectroscopy revealed a largely lipid-lactate peak and mildly elevated choline-creatinine ratio at the margin. Ultimately, he underwent a second craniotomy 5 months after his SRS. The pathology revealed 80% necrotic tumor, with some viable tissue. Among the viable component, small areas of fibrosis, blood pigment and macrophages were seen along with gemistocytic and ganglionic type of cells. The p-53 was rare and there was minimal Ki-67 activity. His post-operative MRI showed no residual necrotic tumor but post-surgical changes. Unfortunately, he developed wound complications and is currently recovering from his third craniotomy.

There are 4 patients who are long-term (>3 years) survivors from glioblastoma, 2 of whom have long-term follow-up after SRS. One patient is alive at 76 months from the diagnosis of glioblastoma in the left temporal lobe, and 75 months from SRS. He is decadron dependent and suffers from neurocognitive decline, slurred speech and expressive aphasia. He was treated with upfront SRS (15 Gy) following 63 Gy of fractionated radiation; he also received a short course of etoposide and >3 years of temozolomide.

A second long term survivor is alive at 50 months from SRS and 76 months from the diagnosis. She suffers short-term memory loss and psychosocial difficulty. She was treated with fractionated radiotherapy (63 Gy) and concurrent temozolomide. At her first tumor recurrence, she was treated with 9 cycles of BCNU & irinotecan. She suffered a second, PET positive, recurrence 9 months later and was treated with SRS (12 Gy). She also was given 28 months of high dose (80 mg) Tamoxifen. Another patient died at 47 months from original diagnosis, and 13 months after SRS, as a result of neurocognitive decline, with apparent local control of his tumor (based on MRI follow-up). He suffered a total of 4 recurrences and was treated quite aggressively with a salvage gross total resection, as well as two separate radiosurgeries at recurrence (20 and 18 Gy at the isocenter, respectively). He also received adjuvant chemotherapy with BCNU, thalidomide and temozolomide at recurrence. In these 3 patients, it is difficult to ascribe the cause of the late treatment effect, which may or may not be attributable to radiosurgery.

A fourth patient is alive at 6 months from SRS and 50 months from diagnosis with recurrent and progressive disease. He was treated with radiation (59.4 Gy) with concurrent and adjuvant temozolomide. He suffered recurrence after 2 years and was treated with bevacizumab and irinotecan initially followed by SRS (10 Gy).

### Outcome

**Figure 1:**
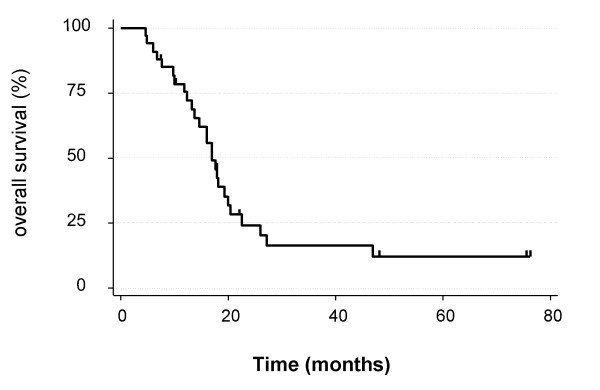
**Overall survival from the initial diagnosis**.

The median survival from the time of initial diagnosis was 16.9 months (4.5 – 76.2 months). The 1-year, 2-year and 3-year overall survival rates were 72%, 23% and 14% respectively. Figure 1 depicts overall survival from the time of diagnosis. There was no difference in survival whether patients received SRS as consolidation or at recurrence (p = 0.4).

**Figure 2:**
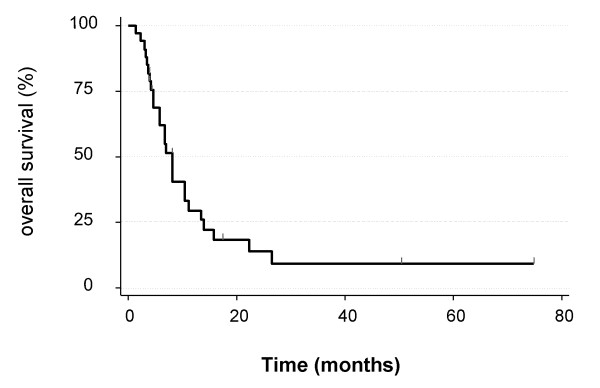
**Overall survival from the time of stereotactic radiosurgery**.

The median survival from the time of SRS was 6.7 months (1.4 – 74.7 months). Figure 2 illustrates the survival curve from the time of SRS. The patients who received up-front SRS had a median survival of 10.3 months compared to 5.3 months among those who received SRS at recurrence, although this difference was not statistically significant on univariate analysis (p = 0.1). Table 3 summarizes the patient survival.

**Table 3:** Patient Survival

	Consolidative SRS (month)	SRS at recurrence (month)	p-value
Median time interval from diagnosis to SRS	1.3	12.1	
Median survival from diagnosis	13.2	17.4	0.4
Median survival from SRS	10.3	5.3	0.1
Median progression free survival from SRS	6.0	3.4	0.04

M = months

SRS = stereotactic radiosurgery

**Figure 3:**
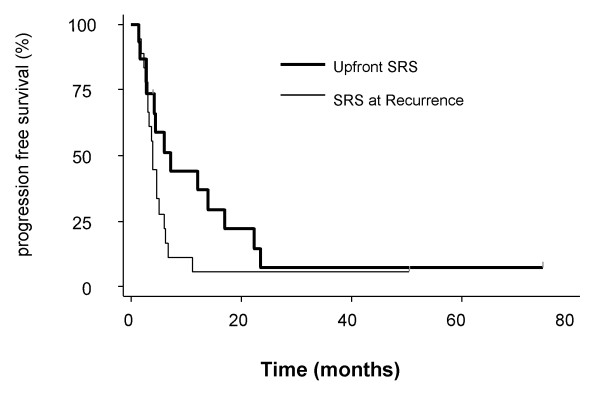
**Progression free survival: upfront stereotactic radiosurgery (SRS) versus SRS for recurrence**.

The median progression free survival from the time of SRS was 4.3 months (1.3 – 74.7 months). On univariate analysis, patients who had SRS as consolidation had a significantly better progression free survival compared to patients who received SRS at the time of recurrence (median progression free survival of 6 months vs. 3.4 months, respectively, p = 0.04) (Figure 3).

As part of out hypothesis-generating analysis, we looked at various prognostic variables for overall survival, including age, tumor location, type of surgery, use of concurrent chemotherapy, and timing of SRS. Only temporal lobe location was significantly better on univariate analysis (p = 0.01).

### Recurrence pattern prior to SRS

For patients (n = 18) who underwent SRS for recurrence, all except one have information about their recurrence pattern prior to the SRS. Nine patients (53%) failed within the previously irradiated volume, while 3 patients (17%) failed both within and at the margin of the previously irradiated volume. Three patients (17%) failed both outside and within the irradiated volume. One patient (6%) failed at the margin only and 1 patient (6%) failed only outside the previously irradiated volume.

### Recurrence pattern after SRS

Seven patients were unable to be evaluated for recurrence, due to loss of follow-up (n = 3) or death prior to imaging follow-up (n = 4). Three patients were locally controlled at last follow-up, 3 of whom are alive at 74.7, 50.4 and 4 months after SRS, and 75.4, 50.4, 7.3 months after initial diagnosis, respectively. Among the 23 patients who experienced recurrence, 21 (92%) recurred within the SRS field or at the field margin, while 1 (8%) recurred outside of the SRS field.

### Salvage treatment

Information about salvage therapy at recurrence was available in 29 patients and is summarized in Table 4. Two patients had SRS for subsequent recurrences and two patients underwent surgical salvage prior to SRS. The majority of patients received salvage chemotherapy.

**Table 4:** Salvage therapy

	Patients treated with upfront SRS, n = 15	Patients treated with SRS at recurrence, n = 18	
	
	Salvage after SRS	Salvage prior to SRS	Salvage after SRS
Surgery	1	2	
Chemo	7	4	14
Radiation		1	
2^nd ^SRS	1		1

## Discussion

glioblastoma continues to be a challenging disease to treat, with most patients succumbing to their disease in the course of a few months to a few years. Since the late 1970s when adjuvant radiation was found to improve survival, very little progress has been made in further improving the survival. In 2005, the EORTC and NCIC conducted a phase III trial, investigating temozolomide during and after radiation [2]; this regimen yielded a modest but significant improvement in survival for this aggressive tumor, a small impact made almost after 25 years since the report by Walker et al established that 60 Gy as the most effective dose for glioblastoma from a pooled retrospective analysis [5].

The inability to control the tumor locally, with distant brain metastases being far less common than local recurrence, led to the hypothesis that by increasing the radiation dose, local control and survival may improve. SRS is one of the methods of dose escalation which had been hypothesized to improve outcome [33,34]. The RTOG 93-05 study randomized >200 patients with glioblastoma to SRS boost in addition to 60 Gy fractionated radiation and BCNU chemotherapy; no survival benefit was appreciated with the addition of an SRS boost [16]. As size was a criterion for eligibility for SRS boost, a subsequent analysis of RTOG data found even by RPA stratification, SRS offers no significant added advantage [35]. In an attempt to further clarify the role of SRS in malignant glioma, Tsao M et al. reviewed the literature and concluded that there is level I-III evidence that the addition of an upfront SRS boost to fractionated radiation and BCNU offers no benefit to survival, local control or quality of life in malignant glioma, with a greater risk of toxicity [36]. Similar conclusions were reached in an evidenced based review in this journal, though the authors conclude that "selected patients may benefit but the specific characteristics of this group have yet to be identified" [1].

Because a high percentage of patients experience local recurrence following standard therapy, salvage therapy is often challenging. Resection, chemotherapy or radiation are the various options which play an important role in the management of recurrent disease [37,38]. Retrospective data, including matched pair analyses suggest that salvage therapy prolongs progression free survival and overall survival, albeit with a selection bias favoring those patients undergoing salvage [39]. Since these tumors are quite infiltrative, a second surgery is often not feasible. Fractionated re-irradiation can be quite risky because a larger volume of previously treated brain is enclosed within the radiated volume. SRS, by virtue of rigid immobilization, allows for minimal radiation dose exposure to surrounding tissue. SRS is therefore well suited for patients undergoing a second course of radiation, as the normally accepted dose tolerances of normal structures would otherwise likely need to be exceeded to ensure adequate target coverage. In the RTOG 93-05 study, 19% of patients randomized to the arm without upfront SRS received SRS as salvage, 6% received fractionated radiation and 35% underwent salvage surgery. In contrast, 6% of patients randomized to the arm with upfront SRS received SRS as salvage, 7% received fractionated radiation and 33% underwent resection. Arguably, one can conclude that the RTOG 93-05 study does not necessarily show that SRS is not beneficial in patients with glioblastoma, but rather SRS can be delayed as part of salvage therapy without a detriment in survival.

The data on salvage SRS for recurrent glioblastoma is sparse and mostly retrospective making it difficult to interpret. A few retrospective studies have examined SRS with recurrent glioblastoma; these are summarized in the Table 5. The median survival after SRS is on the order of 8–12 months. Late toxicity other than radiation necrosis is uncommonly reported. Among those patients who undergo a neurosurgical procedure for progression and/or suspected radionecrosis, necrosis is admixed with viable tumor cells in nearly all samples. Generally, radionecrosis is reported in roughly 5–10% of cases,36,37 though most patients develop radiographic evidence of necrosis [40]. The number of patients who are found to have pathologic necrosis will obviously be impacted by the aggressiveness of the neurosurgeon and the amenability of the tumor and patient to surgical exploration and debulking. As a consequence, the extent of radionecrosis after SRS is difficult to accurately quantify and characterize, but certainly it is not prohibitive to treatment, particularly since tumor progression is the primary means of death and diminished quality of life in most patients.

**Table 5:** Summary or studies on the use of salvage stereotactic radiosurgery for recurrent glioblastoma

**Institution (year)**	**#rGB/# total**	**Median dose [range] (Gy)**	**Prescription**	**MS (M)**	**Late toxicity**
Brigham & Women's Hosp. (1995) [22]	86/86	13 [6-20]	50–90% IDL Median 80%	10	19 pathologic necrosis 1 CN palsy

U. Pittsburgh (1997) [23]	19/107	NR	NR	30	1 symptomatic necrosis 12 pathologic necrosis (of 60 GB patients)

U. Minnesota (1999) [24]	27/46	17 [9-40]	30–90% IDL Median 50%	7	8 pathologic necrosis 6 clinical necrosis

U. Wisconsin (1999) [25]	NR/30	NR	50–80% IDL	7	NR

Cleveland Clinic (2000) [26]	23/23	15 [12-20]	50–80% IDL	10	1 pathologic necrosis 2 increased seizures

UCSF (2002) [27]	14/26	[~10–22]	50% IDL	10	Not assessable

UCSF (2002) [27]	39/54	[~12–18] + marimastat	25–30% IDL	9	Not assessable

U. Heidelberg (2005) [28]	32/32	15 [10-20]	80% IDL covers tumor	10	None

MDACC (2005) [29]	40/40	NR	NR	11	8 pathologic necrosis

Henry Ford (2007) [30]	26/26	NR	18 Gy at margin	9	NR

Current series	18/33	15 [9-20]	80% IDL covers tumor	7	See text

Abbreviations: rGB = recurrent glioblastoma, GB = glioblastoma, SRS = stereotactic radiosurgery; IDL = isodose line. NR = not reported.

In our study, the median survival following SRS was 6.7 months which is comparable with the most reported series. The majority of the patients who received SRS as an upfront boost were treated prior to the reported results of RTOG 95-03. The median survival from the time of SRS for the up-front SRS was slightly better (10.3 months) compared to the patients who had SRS at recurrence (5.3 months). However, the median interval between the diagnosis and SRS was 1.3 months versus 12.1 months for patients who had consolidative SRS and SRS at recurrence, respectively. When we looked at the overall survival from the time of diagnosis, there is no difference in these two groups. Interestingly, we found significant improvement in progression-free survival when SRS was added as a consolidative treatment. We did not analyze the quality of life measure to see if delay in progression free survival is associated with any improvement in quality of life.

We have two long term survivors over 6 years in each group indicating it is not only the treatment but also the tumor biology which is probably crucial. As we develop greater understanding of tumor biology, we might be able to identify a subset of patients who would require more aggressive therapy compared to those who will do better without any aggressive therapy [41,42]. A recent article by Krex et al. suggested that patients who have hypermethylated MGMT protein are the long-term survivors [42]. Hegi et al. reported from the EORTC randomized trial that those patients who had methylated MGMT gene benefited significantly better from temozolomide than the patients who did not [43]. Most of the SRS literature pre-dates the temozolomide era. Currently, whether more aggressive initial local therapy, including SRS, will have more benefit among patients receiving temozolomide, with or without the methylated MGMT gene, is unknown.

In our series, there was no RTOG >grade 2 acute toxicity seen. One patient developed grade 4 late toxicity and underwent craniotomy for this. The pathology largely showed necrosis (80%) with some viable tissue including both fibrosis and tumor cells and minimal Ki-67 activity. Among our 4 long-term survivors who lived over 4 years since the diagnosis of their glioblastoma, three are still alive. One patient died presumably from treatment related toxicity. However, he received aggressive surgery, multiple courses of radiation and prolonged chemotherapy and all these factors might have contributed to the toxicity rather than just radiation. The other patient who is still alive after 6 years has grade 2 late toxicity.

In conclusion, SRS is generally a well tolerated treatment both as a boost and as a salvage therapy. As there was no difference in survival between the two groups, the decision of adding SRS to fractionated treatment should be based on individual patient status and preference. In the temozolomide era, the role of SRS needs to be better defined by future studies.

## Competing interests

The authors declare that they have no competing interests.

## Authors' contributions

TB and MM conceived and designed the study. PO, MS, WP, GEV, KW, DK and MM treated patients on the study. TB, MM and TS analyzed data. All authors participated in drafting and revising the manuscript. All authors have given final approval of the manuscript.
